# Inverse Correlation between Serum Levels of Selenoprotein P and Adiponectin in Patients with Type 2 Diabetes

**DOI:** 10.1371/journal.pone.0034952

**Published:** 2012-04-04

**Authors:** Hirofumi Misu, Kazuhide Ishikura, Seiichiro Kurita, Yumie Takeshita, Tsuguhito Ota, Yoshiro Saito, Kazuhiko Takahashi, Shuichi Kaneko, Toshinari Takamura

**Affiliations:** 1 Department of Disease Control and Homeostasis, Kanazawa University Graduate School of Medical Science, Kanazawa, Ishikawa, Japan; 2 Department of Medical Life Systems, Faculty of Medical and Life Sciences, Doshisha University, Kyotanabe, Kyoto, Japan; 3 Department of Nutritional Biochemistry, Hokkaido Pharmaceutical University, Otaru, Hokkaido, Japan; University of Cordoba, Spain

## Abstract

**Background:**

We recently identified selenoprotein P (SeP) as a liver-derived secretory protein that causes insulin resistance in the liver and skeletal muscle; however, it is unknown whether and, if so, how SeP acts on adipose tissue. The present study tested the hypothesis that SeP is related to hypoadiponectinemia in patients with type 2 diabetes.

**Methodology/Principal Findings:**

We compared serum levels of SeP with those of adiponectin and other clinical parameters in 36 patients with type 2 diabetes. We also measured levels of blood adiponectin in SeP knockout mice. Circulating SeP levels were positively correlated with fasting plasma glucose (*r* = 0.35, *P* = 0.037) and negatively associated with both total and high-molecular adiponectin in patients with type 2 diabetes (*r* = −0.355, *P* = 0.034; *r* = −0.367, *P* = 0.028). SeP was a predictor of both total and high-molecular adiponectin, independently of age, body weight, and quantitative insulin sensitivity index (*β* = −0.343, *P* = 0.022; *β* = −0.357, *P* = 0.017). SeP knockout mice exhibited an increase in blood adiponectin levels when fed regular chow or a high sucrose, high fat diet.

**Conclusions/Significance:**

These results suggest that overproduction of liver-derived secretory protein SeP is connected with hypoadiponectinemia in patients with type 2 diabetes.

## Introduction

We have recently identified selenoprotein P (SeP) as a liver-derived secretory protein that causes insulin resistance and hyperglycemia in patients with type 2 diabetes [1]. SeP (encoded by the *SEPP1* gene in humans) is a plasma protein primarily produced by the liver [2]. SeP, which contains 10 selenocysteine residues per polypeptide, functions as a selenium transport protein [3], [4]; however, its role in glucose homeostasis was unknown. SeP has been emerged from human liver screening for secretory proteins whose hepatic expression levels are significantly correlated with insulin resistance [1]. We have found that blood levels of SeP are elevated in rodents and patients with type 2 diabetes, and that SeP impairs cellular insulin signal transduction and dysregulates glucose metabolism in both hepatocytes and myocytes, at least partly, by inactivating adenosine monophosphate-activated protein kinase (AMPK). These findings suggest that SeP functions as a “hepatokine” causing insulin resistance in the liver and skeletal muscle of patients with type 2 diabetes. However, it was still unclear whether SeP acts directly on adipose tissue or adipocytes.

Adiponectin is an adipocyte-derived secretory protein that improves systemic glucose tolerance and protects the vasculature from atherosclerosis [5], [6], [7], [8]. Circulating levels of adiponectin decrease in patients who are obese, and those with insulin resistance and type 2 diabetes [9], [10]. Additionally, hypoadiponectinemia is an independent risk factor for developing type 2 diabetes and cardiovascular disease [11], [12], [13], [14]. Three different forms of adiponectin have been identified: a high molecular weight (HM) form, a low molecular weight form, and a trimeric form [15], [16]. HM adiponectin possesses the most potent insulin-sensitizing activity and is more closely associated with the development of type 2 diabetes than total adiponectin [17]. Adiponectin gene expression is regulated by multiple transcriptional factors and stimulators including peroxisome proliferator-activated receptor (PPARγ) [18], [19]; however, the molecular mechanisms by which adiponectin production is suppressed in patients with type 2 diabetes are not fully understood.

We hypothesized that SeP acted on adipocytes and affected adiponectin production. To test this, we compared serum SeP levels with those of adiponectin in patients with type 2 diabetes. We also measured blood levels of adiponectin in SeP knockout mice to determine whether SeP contributed to hypoadiponectinemia induced by a high calorie diet.

## Results

### Circulating selenoprotein P is associated with fasting plasma glucose and total and high-molecular adiponectin levels in patients with type 2 diabetes

**Figure 1:**
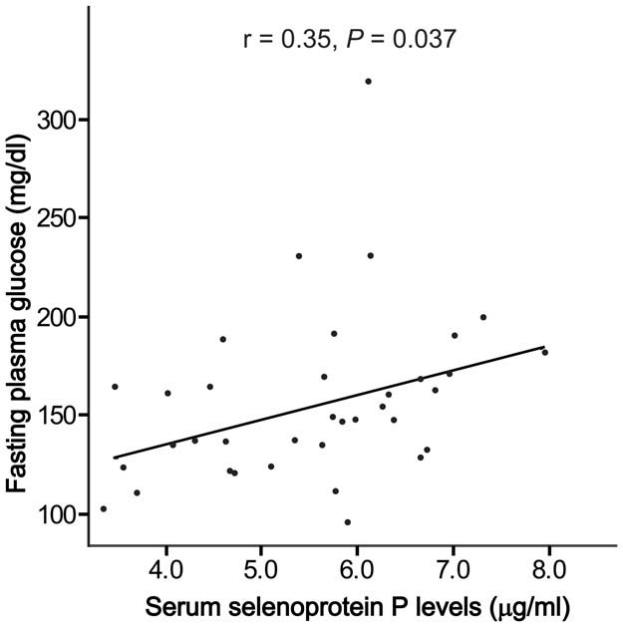
Correlation between selenoprotein P and glucose concentrations in patients with type 2 diabetes. Pearson's analysis r and P values are shown.

**Figure 2:**
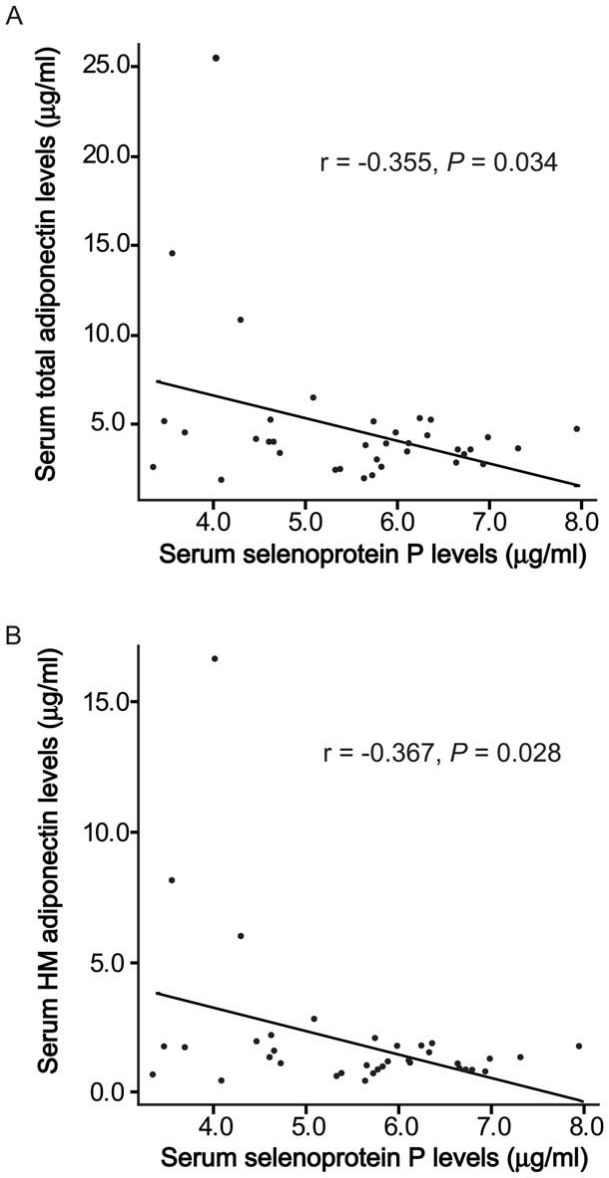
Correlation between serum levels of selenoprotein P and total adiponectin (A) and high-molecular adiponectin (B) in patients with type 2 diabetes. Pearson's analysis r and P values are shown.

The clinical and laboratory variables of the study subjects are shown in Table 1. Circulating SeP levels were significantly correlated with fasting plasma glucose (*r* = 0.35, *P* = 0.037), as we described previously [1] (Fig. 1). Furthermore, serum SeP concentrations were negatively associated with both total and HM adiponectin levels in patients with type 2 diabetes (*r* = 0.355, *P* = 0.034; *r* = 0.367, *P* = 0.028) (Fig. 2). No significant correlation was found between SeP and age, body weight, body mass index, liver function, high-sensitivity C-reactive proteins (hsCRP), or QUICKI (Table 2).

**Table 1:** Clinical and laboratory variables.

Characteristics	
*n*	36
Age (years)	58.4±12.4
Gender (M/F)	26/10
Body weight (kg)	64.3±11.3
BMI (kg/m^2^)	24.5±3.8
Fasting plasma glucose (mg/dL)	154.6±42.5
HbA1c (%)	8.9±2.1
Fasting immunoreactive insulin (µU/mL)	7.06±4.27
QUICKI	0.37±0.05
AST (IU/L)	24.8±16.3
ALT (IU/L)	34.5±34.5
Total cholesterol (mg/dL)	192.1±33.7
Triglyceride (mg/dL)	124.6±46.9
High-sensitivity C-reacitive protein (mg/dL)	0.10±0.10
Selenoprotein P (µg/mL)	5.6±1.2
Total adiponectin (µg/mL)	4.7±4.2
High-molecular weight adiponectin (µg/mL)	1.9±2.6

Data are means±SD. BMI, body mass index; HbA1c, glycosylated hemoglobin; QUICKI, quantitative insulin sensitivity index; AST, aspartate aminotransferase; ALT, alanine aminotransferease.

**Table 2:** Linear regression analysis of selenoprotein P concentrations with clinical parameters.

	SeP concentration	
	*p* value	*r* ^2^
Age	0.431	0.018
Body weight	0.466	0.016
BMI	0.835	0.001
Fasting plasma glucose	0.037*	0.122
HbA1c	0.391	0.022
Fasting immunoreactive insulin	0.424	0.020
QUICKI	0.552	0.010
AST	0.874	0.001
ALT	0.961	0.000
Total cholesterol	0.842	0.001
Triglyceride	0.43	0.018
High-sensitivity C-reacitive protein	0.871	0.0007
Total adiponectin	0.034*	0.126
High-molecular weight adiponectin	0.028*	0.135

* *p*<0.05.

see Table 1 for abbreviations.

### Circulating SeP is an independent predictor of circulating adiponectin levels in patients with type 2 diabetes

We performed a linear regression analysis of HM adiponectin and total adiponectin concentrations to determine clinical factors related to hypoadiponectinemia in patients with type 2 diabetes (Table 3 and Table 4). Serum concentrations of both HM and total adiponectin were negatively associated with age, body weight, QUICKI, and SeP. We generated multivariate linear regression models using HM and total adiponectin as the dependent variable to clarify the contribution of SeP on the decrease in adiponectin concentrations. As shown in Table 5 and Table 6, we found that SeP was a predictor of HM and total adiponectin, independently of age, body weight, and QUICKI.

**Table 3:** Linear regression analysis of high-molecular adiponectin concentrations with clinical parameters.

	High-molecular adiponectin concentration	
	*p* value	*r* ^2^
Age	0.013*	0.168
Body weight	0.035*	0.125
BMI	0.158	0.058
Fasting plasma glucose	0.749	0.003
HbA1c	0.798	0.002
Fasting immunoreactive insulin	0.198	0.051
QUICKI	0.027*	0.135
AST	0.646	0.006
ALT	0.325	0.029
Total cholesterol	0.378	0.023
Triglyceride	0.066	0.096
Total adiponectin	0.0001*	0.99
High-sensitivity C-reacitive protein	0.327	0.029
Selenoprotein P	0.028*	0.135

* *p*<0.05.

See TABLE 1 for abbreviations.

**Table 4:** Linear regression analysis of total adiponectin concentrations with clinical parameters.

	Total adiponectin concentration	
	*p* value	*r* ^2^
Age	0.009*	0.185
Body weight	0.035*	0.124
BMI	0.165	0.056
Fasting plasma glucose	0.728	0.007
HbA1c	0.932	0.0002
Fasting immunoreactive insulin	0.197	0.052
QUICKI	0.030*	0.132
AST	0.557	0.010
ALT	0.250	0.040
Total cholesterol	0.416	0.020
Triglyceride	0.061	0.099
High-molecular adiponectin	0.0001*	0.99
High-sensitivity C-reacitive protein	0.317	0.030
Selenoprotein P	0.034*	0.126

* *p*<0.05.

See TABLE 1 for abbreviations.

**Table 5:** Multivariate regression analysis of high-molecular adiponectin concentration as a dependent variable.

	High-molecular adiponectin concentration	
	β	*p* value
Age	0.310	0.06
Body weight	−0.033	0.848
QUICKI	0.362	0.022*
Selenoprotein P	−0.357	0.017*

* *p*<0.05.

QUICKI, quantitative insulin sensitivity index.

**Table 6:** Multivariate regression analysis of total adiponectin concentration as a dependent variable.

	Total adiponectin concentration	
	β	*p* value
Age	0.337	0.042
Body weight	−0.023	0.891
QUICKI	0.356	0.024*
selenoprotein P	−0.343	0.022*

* *p*<0.05.

QUICKI, quantitative insulin sensitivity index.

### Blood levels of adiponectin are elevated in SeP-deficient mice

**Figure 3:**
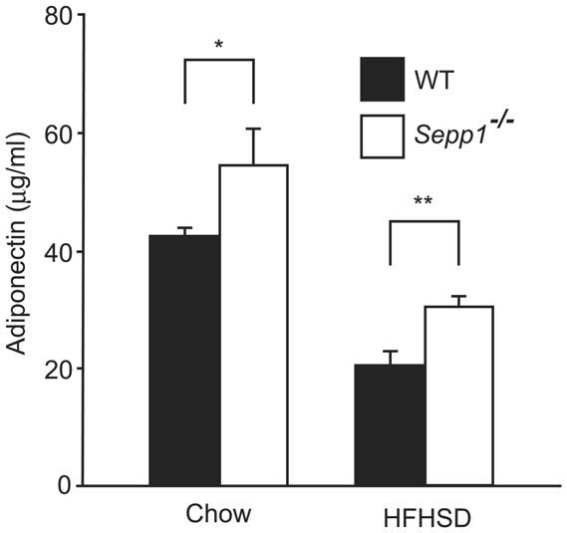
Blood levels of adiponectin in selenoprotein P-knockout and wild-type mice fed regular chow or a high fat, high sucrose diet (n = 5–10). Sixteen week old male mice were fed a high fat, high sucrose diet for 16 weeks. Data represents the means ± SEM. *p<0.05, **p<0.01, vs. wild-type mice.

We measured blood levels of adiponectin in SeP knockout mice fed a high sucrose, high fat diet to examine whether SeP was related to the development of hypoadiponectinemia induced by obesity and diabetes. We previously reported that SeP-deficient mice are protected from insulin resistance and adipocyte hypertrophy induced by a high sucrose, high fat diet, although body weight is unaffected [1]. As shown in Figure 3, SeP-deficient mice exhibited an increase in blood adiponectin levels when fed regular chow and the high sucrose, high fat diet. These results indicate that SeP is connected with the onset of hypoadiponectinemia induced by a high fat, high sucrose diet *in vivo*.

### Expression of genes involved in adipogenesis and inflammation in adipose tissue of SeP knockout mice

**Figure 4:**
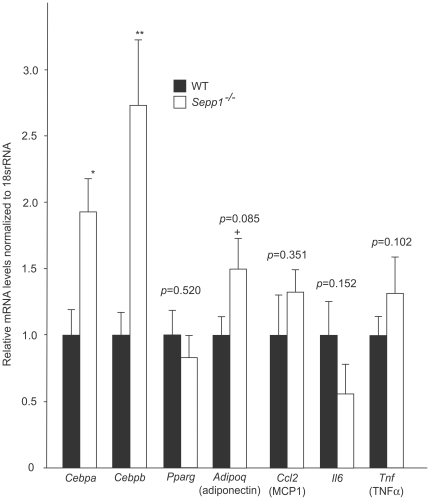
Expression of genes involved in adipogenesis and inflammation in the adipose tissue of selenoprotein P-knockout and wild-type mice fed a high fat, high sucrose diet (n = 5–10). RNA was isolated from epididymal fat of mice. Data represents the means ± SEM. ^+^p<0.1, *p<0.05, **p<0.01, vs. wild-type mice.

We assessed levels of gene expression involved in adipogenesis and inflammation in the adipose tissue of SeP knockout mice fed a high fat, high sucrose diet (Fig. 4). Gene expression levels for CCAAT/enhancer-binding protein-α (C/EBP-α) and CCAAT/enhancer-binding protein-β (C/EBP-β) were up-regulated in SeP-deficient mice. Gene expression levels for adiponectin tended to be increased in the knockout mice. Expression of genes for PPARγ, monocyte chemoattractant protein-1 (MCP1), interleukin-6 (IL-6), and tumor necrosis factor-α (TNF-α) was unchanged.

## Discussion

The results indicate that circulating SeP, a liver-derived secretory protein, is an independent predictor of adiponectin in patients with type 2 diabetes. Furthermore, our findings reveal that SeP-deficient mice were protected from hypoadiponectinemia regardless of nutritional status. These findings lead us to conclude that the increase in circulating SeP is associated with the development of hypoadiponectinemia observed in patients with type 2 diabetes.

We have previously reported that serum levels of SeP in normal subjects and type 2 diabetic patients were 5.1±1.7 and 6.7±0.9, respectively [1]. In the current study, levels of serum SeP in patients with type 2 diabetes were 5.6±1.2 µg/mL (Table 1). The present values were relatively lower than those in our previous report. This might be explained by the fact that the severity of obesity and insulin resistance of the patients in the current study was lower than those in our previous study. More recently, Yang et al. have also shown that serum concentrations of SeP were 3-fold up-regulated in Korean patients with type 2 diabetes compared with normal subjects [20]. The molecular mechanisms by which type 2 diabetes increases blood levels of SeP are not fully understood. However, several lines of evidence has shown that insulin dramatically suppresses the production of SeP in the hepatocytes [1], [21], [22], suggesting that decreased insulin action in the liver may induces the elevation of circulating SeP in patients with type 2 diabetes.

We have previously showed that gene expression levels for SeP in the liver are positively correlated with the severity of insulin resistance measured by glucose clamp experiments in patients with type 2 diabetes [1]. However, in the current study, there were no relationships between serum levels of SeP and the markers of insulin resistance such as QUICKI and fasting immunoreactive insulin. This discrepancy might depend on the fact that the patients in the current study had a low degree of obesity and insulin resistance. Unlike Western subjects, Japanese patients with type 2 diabetes are characterized by decreased insulin secretion rather than insulin resistance. Hence, the clinical markers such as QUICKI, those are estimated by blood insulin levels, might not accurately reflect the sevirity of insulin resistance in these patients. Additional studies by using glucose clamp experiments will provide information of the relationship between serum SeP concentrations and the sevirity of insulin resistance in Japanese patients with type 2 diabetes.

The present study uncovers hyperadiponectinemia in SeP knockout mice fed a high sucrose, high fat diet. In our previous study, SeP knockout mice show an attenuation of adipocyte hypertrophy and an improvement of glucose tolerance and insulin resistance when on a high fat, high sucrose diet [1]. However, the notable finding in the current study was that blood levels of adiponectin in SeP knockout mice fed a high sucrose, high fat diet did not completely reach to those in wild-type mice fed a normal chow. In addition, the present findings in humans showed that the correlation between SeP and adiponectin is not strong, explaining only 13% of the variance in adiponectin levels. These results strongly suggest that the contribution of SeP on hypoadiponectinemia is only partial, and that other factors except the elevation of circulating SeP also participate in the development of hypoadiponectinemia in type 2 diabetes.

More recently, Schoenmakers et al. reported an elevation in blood adiponectin and enhanced insulin signaling in patients with markedly reduced expression of plasma SeP due to a genetic defect in the *SECISBP2* gene that encodes selenocysteine insertion sequence-binding protein 2 [23]. These patients have compound heterozygous *SECISBP2* defects that result in diminished synthesis of most known selenoproteins, such as SeP. We cannot exclude the possibility that defects in selenoproteins other than SeP contributed to the phenotypes in these patients, but their metabolic findings were in good agreement with those in SeP knockout mice. Thus, we believe that the reduction in plasma SeP contributes, at least in part, to the hyperadiponectinemia observed in these patients with a genetic defect in the *SECISBP2* gene.

Growing evidence indicates that type 2 diabetes and hypoadiponectinemia are associated with low-grade inflammation, especially in the adipose tissue [24]. However, the current study showed no correlation between SeP and hsCRP, a representative marker of low-grade inflammation in Japanese patients with type 2 diabetes. Moreover, gene expression levels involved in inflammation were unchanged in the adipose tissue of SeP knockout mice. These results suggest that SeP is not strongly connected with low-grade inflammation observed in type 2 diabetes. More recently, however, Yang et al. have reported that blood levels of SeP are positively and strongly correlated with those of hsCRP in Korean people with type 2 diabetes [20]. The discrepancy between Yang's report and our study might be associated with the fact that our patients in Japan had lower degree of obesity, insulin resistance, and inflammation. Further investigation is necessary to elucidate the action of SeP on low-grade inflammation in the adipose tissue.

A limitation of our study was that we did not measure blood concentrations of selenium in our patients. Several lines of evidence indicate that selenium supplementation increases SeP blood levels [25], [26], [27]. Furthermore, serum selenium levels are positively correlated with SeP levels in humans [28], [29] Additional studies on a larger number of samples are needed to clarify the connections among selenium, SeP, and adiponectin.

In conclusion, our results suggest that the elevation of hepatokine SeP is connected with hypoadiponectinemia in type 2 diabetic conditions. Further cellular or animal experiments are needed to investigate whether SeP directly acts on the adipocytes or adipose tissue.

## Materials and Methods

### Ethics Statement

All patients provided written informed consent for this study. The experimental protocol was approved by the Ethics Screening Committee of Kanazawa University Hospital (Approval NO. 1123), and the study was conducted in accordance with the Declaration of Helsinki.

The animal study was carried out in accordance with the Guidelines on the Care and Use of Laboratory Animals issued by Kanazawa University. The protocol was approved by the ethical committee of Kanazawa University (Approval NO. 50202). All surgery was performed under sodium pentobarbital anesthesia, and all efforts were made to minimize suffering.

### Human study

Blood samples were obtained from 36 patients with type 2 diabetes, who were admitted to Kanazawa University Hospital from April 2006 to March 2007. Some patients in our previous study [1] were involved in the current study. Patients with type 2 diabetes were diagnosed based on criteria established by an expert committee on the diagnosis and classification of diabetes mellitus. Patients were treated with diet therapy alone or with sulfonylureas or insulin; no patient received any other oral hypoglycemic agents, such as pioglitazone or a biguanide. Furthermore, participants receiving statins, angiotensin-converting enzyme inhibitors, or angiotensin II receptor blockers were excluded. After an overnight fast, venous blood samples were withdrawn from each patient.

### Assays

Serum SeP levels were measured by ELISA using two monoclonal antibodies, as described previously [30]. Serum levels of total and HM adiponectin were measured using ELISA kits purchased from Ostuka (Tokyo, Japan) and Fujirebio (Tokyo, Japan), respectively. The immunonephelometric method was used to measure high-sensitivity C-reactive protein (hsCRP). The immunoenzymometric assays used for quantifying insulin were conducted with kits purchased from Tosoh (Shunan, Japan). The quantitative insulin sensitivity index (QUICKI) was used as a convenient index of insulin resistance [31]. QUICKI values were calculated using the following formula:



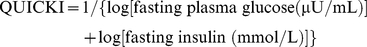
Mouse study

*Sepp1*-deficient mice were produced by homologous recombination using genomic DNA cloned from a Sv-129 P1 library, as described previously [3]. *Sepp1*-deficient mice were backcrossed to C57/BL6 for up to three generations. Sixteen-week-old male mice were fed a high fat, high sucrose diet for 16 weeks (n = 5–10). After a 12-h fast, blood samples were obtained. The high fat, high sucrose diet (D03062301) was purchased from Research Diets (New Brunswick, NJ, USA). *Sepp1*-deficient mice experiments were performed using frozen blood samples adipose tissue obtained from our previous study [1]. Serum adiponectin levels were determined using a mouse adiponectin ELISA kit (Otsuka), according to the manufacturers' instructions.

### Real-Time Quantitative Polymerase Chain Reaction

Total RNA was extracted from each epididymal adipose tissue using the RNeasy Lipid Tissue Mini Kit (Qiagen). cDNA was synthesized from 100 ng of total RNA using a high-capacity cDNA Archive Kit (Applied Biosystems). Real-time quantitative Polymerase Chain Reaction (PCR) was performed for C/EBP-α, C/EBP-β, PPARγ, adiponectin, MCP-1, IL-6, and TNF-α, using mRNA using the ABI Prism 7900 Sequence Detection System (Applied Biosystems) as previously described [32]. The sets of primers and TaqMan probes were proprietary to Applied Biosystems (TaqMan Gene Expression Assays product). To control for variation in the amount of DNA available for PCR in the different samples, gene expression of the target sequence was normalized in relation to the expression of an endogenous control, 18S rRNA (TaqMan Control Reagent Kit; Applied Biosystems). The PCR conditions were one cycle at 50°C for 2 min, 95°C for 10 min, followed by 50 cycles at 95°C for 15 s and 58°C for 1 min.

### Statistical Analysis

Data are shown as means ± SDs for the human study, unless stated otherwise. The relationship between individual variables was analyzed by Pearson's simple correlation, and multiple regression using a forced entry manner. We report squared correlation coefficients and *P* values for the univariate linear regression analysis and coefficients and *P* values for the multiple linear regression analysis. Data are shown as means±SEs for the mouse studies. Data involving more than two groups were assessed by analysis of variance. The Japanese Windows edition of SPSS (ver. 11.0; SPSS, Inc., Chicago, IL, USA) was used for statistical analyses. *P* values<0.05 were considered to indicate statistical significance.
